# Supplementary material for: Determinants of solid fuel use in Sub-Saharan Africa: A multilevel analysis using DHS data
Source: PLoS One. 2025 Apr 25;20(4):e0321721. doi: 10.1371/journal.pone.0321721 (PMC12027170; doi:10.1371/journal.pone.0321721)
Supplement: Attachment — Submitted filename: Response to Reviewers.docx [file pone.0321721.s002.docx]

**Point-by-point response to editor and reviewers**

Please accept our revised manuscript and note our point-by point response to reviewer below for the manuscript titled **"** **PONE-D-24-55055, Analyzing Fixed and Random Effects on Solid Fuel Use in Sub-Saharan Africa: Multilevel Mixed-Effects Modeling Approach with 29 DHS Data**

**PLOS ONE "**

**Note:** Responses for each issue raised by Academic Editor and Reviewers indicated by highlight.

**Authors’ response:** Thank you dear editor, we have seen all your concerns and done as per you recommendation.

**Reviewer Comments:
Reviewer #1**

Reviewer #1: General Comment:
The study addresses an important public health and environmental issue- solid fuel in Sub-Saharan Africa. The methodological approach is appropriate to the nature of the data and uses multilevel mixed-effects modeling, taking into consideration the hierarchical structure of the data. The dataset included 29 DHS surveys for comprehensive regional coverage, hence a large sample size of 233,391 households.

However, the manuscript requires thorough proofreading. Poor grammar and awkward phrasing significantly detract from the content of the manuscript; for example, "Since the human being exists on land.". The findings lack a strong connection with the existing literature. Comparisons are made with other regions, but implications for local policy and practice development are not well developed. The inclusion of P-values along with the AOR is unclear in some sections and needs further explanation. The abstract has failed to provide a clearly articulate the research question and the novelty of this study. The term "Mixed and Random Effects" in the title is ambiguous and might be puzzling for some readers. Whereas it was able to identify factors influencing solid fuel use, it has not indicated how the findings could provide useful information for specific interventions or policies.

**Authors’ response:** Thank you very much dear reviewer for your time and effort for showing authors gaps and for constructive comments to be corrected. Based on these comments authors addressed all issues raised.

Detailed Comments:
**Title:**
01. The title could be simplified for clarity, e.g., “Determinants of Solid Fuel Use in Sub-Saharan Africa: A Multilevel Analysis Using DHS Data.”

**Authors’ response:** we thank for this comment and corrected as recommended.

02. The term "Mixed and Random Effects" in the title is ambiguous and might be puzzling for some readers.
**Authors’ response:** Sorry for such inconvenient but now corrected.

**Abstract:**
03. The first sentence of the abstract is unnecessarily broad.

**Authors’ response:** Authors thank for this comment and we have rephrased this sentence.

04. The abstract has failed to clearly articulate this study's research question and novelty.

**Authors’ response:** Thank you very much dear reviewer, authors rephrased this section.

05. The aim of the study written in the abstract is not clear.

**Authors’ response:** Authors thank you the reviewer and we rephrased.
06. Emphasize actionable insights and policy recommendations in the conclusion of the abstract.
**Authors’ response:** Authors appreciate the reviewer issues and we corrected as per your recommendation.

**Introduction**:

07. The introduction highlights the problem very well but does not provide a clear articulation of the research gap. For instance, while it mentions previous studies on small samples or in specific regions, how this larger dataset adds value needs to be better stated. Citations are irregularly formatted and need to follow the journal's formatting rules.
**Authors’ response:** we thank you for the very valuable comments in this regard and authors as corrected as suggested by the reviewer.

**Methods:**
08. The multilevel modeling approach has been appropriately described, though model selection and handling of missing data need further clarity.

**Authors’ response:** Thank you very much dear reviewer for this comment. We revised this section.
09. On ethics approval, it says, "Not applicable" since the data used are secondary; however, explaining in detail, how the DHS protects anonymity and adheres to the ethical requirements would strengthen this section.
**Authors’ response:** Thank you very much for the comment. We accept the comment and the authors included the reviewer's idea.

10. The size and scope of the data used in the manuscript require an explanation of how missing data were treated.

**Authors’ response:** Thank you very much dear reviewer for this comment. We revised this section.
11. Although many variables are represented, there is little explanation of why certain predictors were chosen and how they relate to the existing theories or frameworks on solid fuel use. The operationalization of the variables, particularly those at the community level, is not fully described about poverty and education levels.

**Authors’ response:** Thank you very much for the issues. The proportion of households is categorized as low poverty level (those with ≥ 50%) and higher poverty level (those with <50%) using the national median value. As well as community-level education, the same way as the poverty level is defined. Therefore, we included it in the manuscript.

**Results:**
12. The prevalence of the use of solid fuel is presented quite clearly; tables and figures should be better formatted for readability.

**Author’s response:** We, authors would really thank for pointing us such very relevant comments and these were paraphrased.

13. Results must integrate findings into contextual interpretations and not merely state statistical outputs. For instance, the discussion of the Adjusted Odds Ratios (e.g., AOR = 12.46 for poor households) could include real-world implications.

**Authors’ response:** Thank you very much for the comment. Corrected as suggested by the reviewer.

14. Table heads and figure captions should be intelligible independently.
**Authors’ Response:** Thank you for your insightful comment. We have revised the table heads and figure captions to ensure they are self-explanatory.

15. The magnitude of the odds ratios seems plausible, but the authors could interpret the outliers or very high AOR values-for example, AOR = 144.49 for community-level poverty-more critically. Such extreme values are indicative of highly stratified populations or possibly overlapping variables.

**Authors’ Response:** Thank you for your valuable feedback and authors’ include possible reason.
16. The AUC is well described but could be briefly explained for readers who may not be familiar with this measure.

**Authors’ response:** we thank for this appreciated comment to these issues and we modified as per suggested by the reviewer.

17. The odds ratio for low-income communities seems very high (AOR = 144.49), questionable about the granularity of the poverty measure. The author should check the causes.

**Authors’ Response:** Thank you for your valuable feedback and authors’ include possible cause of for such high AOR.
18. The study has shown that even the most complete model- Model III-explains only a small proportion of the variance ICC = 0.37. the authors should present possible unmeasured variables.
**Authors’ Response:** Thank you for your insightful comment and authors included possible unmeasured variables.

**Discussion:**
19. While the discussion mentions broad recommendations, it lacks specificity. The authors miss an opportunity to provide evidence-based, region-specific recommendations for policymakers.
**Authors’ Response:** Thank you for your valuable feedback. We believe this will strengthen the overall impact of our work and provide actionable insights for local decision-makers. Therefore, the authors include this pertinent factor.

20. While the discussion mentions broad recommendations, it lacks specificity.

**Authors’ Response:** We appreciate the reviewer’s feedback and we enhanced the discussion by including specific, actionable recommendations tailored to the study findings to provide greater clarity and practical value.

21. While the authors recognize the structural and cultural barriers, actionable recommendations-such as specific programs or policies aimed at reducing dependence on solid fuels are in need of more emphasis in this section.

**Authors’ Response:** We have incorporated these recommendations in the revised section to ensure actionable and context-specific solutions are adequately emphasized. We trust this enhancement strengthens the manuscript and aligns with your suggestions.

22. Authors should critically review the limitations regarding the use of secondary data from DHS, including possible biases and inability to control the selection of variables.
**Authors’ Response:** Thank you for the insightful comment. We acknowledge that using secondary data from the Demographic and Health Surveys (DHS) has inherent limitations, which we have now addressed more explicitly in the revised manuscript.

23. The reported prevalence of solid fuel use is identified and described in the result section of this study which befits the large dataset that this study leveraged. Nevertheless, the presentation might have been contextual, for instance, comparing it with certain countries outside of Sub-Saharan Africa or global averages.

**Authors’ Response:** Thank you for your thoughtful suggestion. We appreciate your emphasis on contextualizing the reported prevalence of solid fuel use. While the current study primarily focuses on trends and determinants of solid fuel use within Sub-Saharan Africa (SSA), we agree that comparing these findings with countries outside SSA or global averages would provide additional valuable context. We believe this addition will enhance the contextual value of the study and appreciate your valuable input in improving our work.

24. While household heads of older age were more likely to use solid fuel, it was specifically with the categories of age >60 years, AOR = 1.12. The study speculates on generational differences in energy preferences; however, the discussion fails to provide empirical evidence or citations to support this claim.

**Authors’ Response:** Thank you for this interesting comment we included this revision this revision to address the reviewer’s concerns.

25. The authors miss an opportunity to provide evidence-based, region-specific recommendations for policymakers.

**Authors’ Response:** we thank you dear reviewer, accept the suggestion, and included region-specific, evidence-based recommendations for policymakers in the revised manuscript.

26. Through the discussion mentioned the findings of other studies however, it misses the comparison or contrast of findings.

**Authors’ response:** Authors thank you dear reviewer, we have compared it with previous studies even if it includes are country, but put as a limitation

27. Whereas the authors have focused on individual-level predictors, community-level factors i.e., poverty and education remain underexplored in the discussion. The discussion may be further strengthened by exploring the dynamics of how community-level factors shape or influence individual decisions.

**Authors’ response:** Authors thank you, dear reviewer; we have included strengthening the manuscript.
28. While limitations are recognized, they are only superficially discussed. The study does not discuss the possible bias from using self-reported data or the limitations of secondary data. The greatest limitation is the cross-sectional nature of the analysis, which should be discussed more critically. The longitudinal data might have shown causality, which is missing here.

**Authors’ Response:** Thank you dear reviewer, we have incorporated these limitations in the revised manuscript.
**Conclusion:**
29. The conclusion summarizes the findings appropriately but should highlight the study's novelty and its practical implications, such as recommendations for policymakerss.

**Authors’ Response:** we thank you, dear reviewer, accept the suggestion, and include region-specific, evidence-based recommendations for policymakers in the revised manuscript.

Overall comment:
The manuscript needs a lot of work in terms of writing quality, contextualization of findings, and depth of discussion.

**Authors’ Response:** Thank you for your feedback; I will revise the manuscript to improve the writing quality, better contextualize the findings, and deepen the discussion.

**Reviewer #2: Following are my comments regarding the paper:**
1) The title of the paper seems misleading, as the primary focus appears to be on the determinants of solid fuel usage in sub-Saharan Africa (SSA). The title should better reflect the main objectives and scope of the study.

**Authors’ Response:** We appreciate the reviewer's comment and have revised the title to more accurately reflect the study's primary focus on the determinants of solid fuel usage in Sub-Saharan Africa.

2) The introduction/background and contribution/value addition of that study fail to generate significant interest. The authors should present more compelling points to address these problems.

**Authors’ Response:** Thank you for your comment and we have revised the introduction to emphasize the significance of the study by clearly outlining the research gaps, highlighting its innovative approach, and demonstrating its potential impact in addressing the identified problems.

3) The paper is missing a literature review section. The authors should discuss prior studies in the field, identify gaps in the existing literature, and justify how their approach addresses these gaps. This will provide a stronger foundation for the research.

**Authors’ Response:** We sincerely thank the reviewer for their valuable feedback and insightful comments, which have significantly improved the quality and clarity of our manuscript. So we have revised this part.

4) The rationale for using the multilevel logistic regression model is not sufficiently explained. The authors should provide a thorough justification for why this model is appropriate for the study, supported by relevant literature.

**Authors’ Response:** Thank you for this valuable comment and we have clarified this in the revised manuscript.

5) The Demographic and Health Surveys (DHS) were conducted in different years for various countries in SSA. The authors should clearly explain how they combined data from different households and years into a single dataset. They must address potential issues that may arise from merging datasets of varying sizes and discuss why this approach does not compromise the validity of the analysis.

**Authors’ Response:** We appreciate the reviewer's comment and have addressed this concern.

6) The authors state, “Variables with a P-value <.25 in the bivariate analysis were selected for multivariate analysis.” This threshold requires further explanation and justification, as it is not a standard practice in most studies.

**Authors’ Response:** Thank you for highlighting this point. We have now added relevant references to justify this approach and included a more detailed explanation in the manuscript.
7) The study primarily relies on a multilevel logistic regression model. It is essential to perform robustness checks using at least one alternative, more advanced statistical model to validate the baseline findings and ensure their reliability.

**Authors’ Response:** We appreciate the reviewer's insightful comment regarding the need for robustness checks using an alternative statistical model. In this study, we primarily relied on a multilevel logistic regression model, as it is well-suited for addressing the hierarchical structure of the data. To ensure the validity of our findings, we conducted rigorous model diagnostics, including the Akaike Information Criterion (AIC), Bayesian Information Criterion (BIC), deviance, and the area under the Receiver Operating Characteristic (ROC) curve, which are widely recognized measures of model performance and fit. These metrics provide robust evidence for the reliability of our results. Moreover, similar studies in the field have consistently relied on such diagnostics to validate their findings without employing alternative advanced models. While we acknowledge the value of additional robustness checks, attempting to incorporate an alternative statistical model would necessitate significant changes to the entire analytical framework, potentially shifting the focus of the study. We believe the current approach is comprehensive and appropriate for addressing the research objectives.
8) The policy implications section should be expanded, offering detailed and actionable recommendations based on the findings. Additionally, the paper should discuss the study’s limitations and suggest directions for future research to address any unresolved issues.

**Authors’ response:** Authors thank you dear reviewer, we have revised to strengthen the manuscript our research work including previous studies
**Authors’ response:** we believed that we have addressed all the issues raised the by dear editor and reviewers sufficiently. If there are additional issues the reviewer expect to be addressed, we encourage further elaborating the issues. Thank you one again for valuable comments raised, knowledge sharing and for your time to help us.
