# Supplementary material for: Determinants of solid fuel use in Sub-Saharan Africa: A multilevel analysis using DHS data
Source: PLoS One. 2025 Apr 25;20(4):e0321721. doi: 10.1371/journal.pone.0321721 (PMC12027170; doi:10.1371/journal.pone.0321721)
Supplement: Attachment — Submitted filename: Response to Reviewer12.docx [file pone.0321721.s004.docx]

A point-by-point response to editor and reviewers

Please accept our revised manuscript and note our point-by-point response to the reviewer below for the manuscript titled “PONE-D-24-55055R2

Determinants of Solid Fuel Use in Sub-Saharan Africa: A Multilevel Analysis Using DHS Data

PLOS ONE”

**Note:** Responses for each issue raised by the Academic Editor and Reviewers are indicated by highlight.

**Authors’ response:** Thank you, dear editor. We have seen all your concerns and have done as per your recommendation.

Reviewer #1: The authors have made significant revisions based on reviewer feedback, improving the structure, clarity, and scientific rigor of the study. However, the few revisions commented on below will further enhance the quality of this manuscript.

Comments:
01. The manuscript still contains awkward phrasing and grammatical errors, so it requires thorough proofreading by a native speaker.

**Authors’ response:** Thank you very much, dear reviewer, for your comments that were able to fill the authors' gaps. Therefore, the authors rephrased and edited the whole document.

02. There is still considerable ambiguity surrounding the display of P-values and Adjusted Odds Ratios (AOR). Explain briefly why the odds ratios for some factors are so high or low. Describe how model selection was influenced by AIC, BIC, and ICC values.

**Authors’ response:** Thank you very much, dear reviewer. We have explanations of P-values along with the AOR from line numbers 154 to 169.

03. The policy suggestions are still general and wide-ranging. Provide evidence-based, nation-specific solutions to reinforce the policy implications.

**Authors’ response:** Authors thank you, dear reviewer; we have revised from line number 366.
**Authors’ response:** We believe that we have addressed all the issues raised by the dear editor and reviewers sufficiently. If there are additional issues the reviewer expects to be addressed, we encourage further elaboration on the issues. Thank you once again for the valuable comments raised, the knowledge sharing, and your time to help us.
